# Diabetic rats present higher urinary loss of proteins and lower renal expression of megalin, cubilin, ClC‐5, and CFTR

**DOI:** 10.14814/phy2.13335

**Published:** 2017-07-04

**Authors:** Miriam F. Figueira, Raquel C. Castiglione, Carolina M. de Lemos Barbosa, Felipe M. Ornellas, Geórgia da Silva Feltran, Marcelo M. Morales, Rodrigo N. da Fonseca, Jackson de Souza‐Menezes

**Affiliations:** ^1^ Laboratório Integrado de Ciências Morfofuncionais Núcleo em Ecologia e Desenvolvimento Socioambiental de Macaé Universidade Federal do Rio de Janeiro Macaé Rio de Janeiro Brazil; ^2^ Laboratório de Fisiologia Celular e Molecular Instituto de Biofísica Carlos Chagas Filho Universidade Federal do Rio de Janeiro Rio de Janeiro Brazil; ^3^ Instituto de Biologia Roberto Alcântara Gomes Universidade do Estado do Rio de Janeiro Rio de Janeiro Rio de Janeiro Brazil

**Keywords:** CFTR, ClC‐5, cubilin, diabetes, endocytosis, kidney, megalin, proximal tubule

## Abstract

Diabetic nephropathy (DN) occurs in around 40% of those with diabetes. Proteinuria is the main characteristic of DN and develops as a result of increased permeability of the glomerulus capillary wall and/or decreased proximal tubule endocytosis. The goal of this work was to evaluate renal function and the expression of megalin, cubilin, CFTR (cystic fibrosis transmembrane conductance regulator), and ClC‐5 in the proximal tubule and renal cortex of rats with type 1 diabetes. Male Wistar rats were randomly assigned to control (CTRL) and diabetic (DM) groups for 4 weeks. Renal function was assessed in 24‐h urine sample by calculating clearance and fractional excretion of solutes. The RNA and protein contents of ClC‐5, CFTR, megalin, and cubilin were determined in the renal proximal tubule and cortex using real‐time polymerase chain reaction and western blotting techniques, respectively. The results showed higher creatinine clearance and higher urinary excretion of proteins, albumin, and transferrin in the DM group than in the CTRL group. Furthermore, the renal cortex and proximal tubule of diabetic animals showed downregulation of megalin, cubilin, ClC‐5, and CFTR, critical components of the endocytic apparatus. These data suggest dysfunction in proximal tubule low‐molecular‐weight endocytosis and protein glomerulus filtration in the kidney of diabetic rats.

## Introduction

Diabetic nephropathy (DN) is a disabling kidney disease and is one of the most frequent and lethal complications of diabetes, occurring in 30–40% of patients (Gross et al. 2005; Pugliese 2014). DN is also the biggest contributor to chronic kidney disease and the increased requirement for dialysis. Different therapies are being studied with the aim of improving treatment for this condition. The use of stem cells and microRNA technology are strong candidates, but, despite intensive efforts, new strategies are not yet in widespread use and a significant number of patients with DN are still reaching end‐stage renal disease (Alberti and Zimmet 1998; Zimmet et al. 2001; Castiglione et al. 2013; Abdel Aziz et al. 2014; Figueira et al. 2014; Pugliese 2014; Kato and Natarajan 2015). Diabetic kidney pathology, especially the initial phase, is not completely understood and more research in this field is relevant to prevent renal deterioration and to reduce mortality in patients with diabetes.

In humans, the most commonly observed features of DN are microalbuminuria (excretion of albumin in urine between 30 mg dL^−1^ and 300 mg dL^−1^ per day), glomerular basement membrane thickening accompanied by mesangial matrix expansion, and tubulointerstitial fibrosis (Zimmet et al. 2001; Gross et al. 2005). The search for markers of early DN has been intense in recent decades, and the presence of microalbuminuria is one of the most studied predisposing factors; this is also used in clinical practice for diagnosing kidney disease (Gross 2003; Jerums et al. 2009).

In patients with DN, microalbuminuria and proteinuria are known to be mainly subsequent to glomerular injury, which is generated by abnormalities in the glomerular endothelium and increased permeability of the capillary wall, thereby causing the loss of high‐molecular‐weight proteins (proteins with molecular weight >65 kDa) and albumin in urine. However, these changes alone do not completely explain how patients develop proteinuria, and proximal tubule injury is now emerging as a critical mechanism that has not been fully elucidated yet. Recently, the proximal tubule has received considerable attention in DN pathophysiology and several studies suggest that this part of the nephron is a primary target, and not a bystander, of various deleterious factors such as reactive oxygen species and advanced glycation end products (Phillips and Steadman 2002; D'Amico and Bazzi 2003; Thomas et al. 2005).

Under physiological conditions, the proximal tubule has an important role in the reabsorption of filtered low‐molecular‐weight (LMW) proteins (proteins with molecular weight <65 kDa) and albumin. Proximal tubule cells reabsorb these proteins via a complex mechanism of receptor‐mediated endocytosis that involves ion‐channel‐mediated acidification of the endocytic vesicle as a key step. Two multiligand receptors, megalin and cubilin, are involved in this process. ClC‐5, a Cl^−^/H^+^ exchanger, and CFTR (cystic fibrosis transmembrane conductance regulator), a Cl^−^ channel, are both involved by maintaining the acidification of the endocytic vesicle, a key step for protein reabsorption at the proximal tubule cells (Zhai et al. 2000; Barth and Argraves 2001; Marshansky et al. 2002; Christensen et al. 2003; Jentsch 2005; Jentsch et al. 2005; Carraro‐Lacroix et al. 2010). Nonetheless, functioning of the proximal tubule endocytic machinery is still poorly understood in the diabetes context.

The goal of this work was to determine if type 1 diabetes induces low expression of megalin, cubilin, ClC‐5, and CFTR in renal tissues and if this fact is associated with the microalbuminuria and LMW proteinuria observed in DN disease. With this aim, we analyzed the renal function and the mRNA plus protein content of the receptors and transporters involved in the endocytic machinery in kidney cortex and proximal tubule of type 1 diabetic rats.

## Methods

### Animal model

This study was approved by the Ethics Committee on animal use at the Health Sciences Centre, Federal University of Rio de Janeiro (protocol number 01200.001568/2013‐87). Animals were obtained from Animal Service (Rat Section), Health Sciences Center, Federal University of Rio de Janeiro. All animals received humane care in compliance with the guide prepared by Brazilian Society of Animal Laboratory Science and National Council for the Control of Animal Experimentation.

Wistar rats (180–230 g, 8–9 weeks old) were housed in a 12 h:12 h light:dark facility at a temperature of 22 ± 2°C with food and water provided ad libitum. No more than three animals were kept per cage during the experiments.

### Experimental design

Rats were randomly assigned into two groups: a control group (CTRL; *n *=* *6) and a diabetic group (DM; *n *=* *8). In CTRL rats, an intraperitoneal injection of 0.5 mL of vehicle (0.1 mol/L sodium citrate buffer, pH 4.7) was administered on the first day of the experiment; in DM rats, an intraperitoneal injection of 45 mg kg^−1^ body weight of streptozotocin (STZ) (Sigma, S0130) dissolved in 0.5 mL of vehicle was administered. The rats were considered diabetic when 5‐h fasting blood glucose levels exceeded 250 mg dL^−1^.

**Figure 1:**
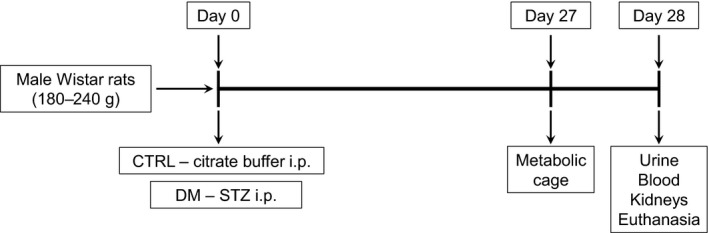
Timeline representation of the experimental design. Male Wistar rats weighing between 180 and 240 g were divided into two groups: control (CTRL) and diabetes mellitus (DM). The CTRL group received an intraperitoneal injection of 0.1 mol/L citrate buffer (pH 4.7) and the DM group received an intraperitoneal injection of streptozotocin (STZ) 45 mg kg^−1^ body weight. After 4 weeks, both groups were killed for blood and kidney collection; 24 h before that, the animals were placed in a metabolic cage, which allowed urine collection and measurement of renal function.

Four weeks after induction of diabetes or vehicle administration, the rats were placed in metabolic cages (Tecniplast, Italy), for 24‐h urine collection, following anesthesia and sedation with an intraperitoneal injection of ketamine (100 mg kg^−1^ body weight; Koning) and xilasin (10 mg kg^−1^ body weight; Bayer). The animals were killed by inducing hypovolemic shock during blood collection by cardiac puncture (Fig. 1).

### Blood glucose and body weight measurements

From day 0 until day 28, blood glucose and body weight were assessed twice a week. For blood glucose measurements, all animals were fasted for 5 h. A drop of tail blood was taken by making a small snip in the terminal 1.5 mm of the tail with a scalpel. Glucose concentration in the blood was determined using a OneTouch Ultra glucose meter (LifeScan, Wayne, PA, USA). The animals were weighed using a precision balance (Souza‐Menezes et al. 2007).

### Analysis of metabolic data for assessing renal function

Twenty‐four hours before they were killed, all animals were placed in individual metabolic cages with free access to water and food for 19 h and fasting for the last 5 h with free access to water. Urine was collected for 24 h and water ingestion was measured during the same period (the amount of water left in the drinking fountain was subtracted from the initial amount added). The blood glucose concentration was determined in tail blood (see above), followed by deep anesthesia and sedation (see above). Total laparotomy was performed for blood collection and kidney excision. Using a 1‐mL syringe, previously rinsed with 0.1 mol/L EDTA, 1 mL of blood was collected by puncturing the inferior vena cava and placed on ice immediately. Blood was centrifuged at 5000*g* for 5 min at 4°C to separate the plasma.

The concentration of the following solutes was determined in urine and/or plasma: creatinine (kinetic assay; Gold Analisa, Brazil), glucose (enzymatic colorimetric assay; Gold Analisa, Brazil), total protein (colorimetric assay; Gold Analisa, Brazil), sodium (colorimetric assay; InVitro, Brazil), chloride (colorimetric assay; Doles, Brazil), and potassium (colorimetric assay; Doles, Brazil). Total protein and albuminuria were measured in the urine samples of CTRL and DM animals using a colorimetric assay based on pyrogallol red (Gold Analisa, Brazil) and an immunoturbidimetric method (Gold Analisa, Brazil), respectively.

The fractional excretion of solutes was determined; this represents the percentage of solute excreted in the urine from glomerular filtration. Fractional excretion was estimated using the following equation: FE_s_ = (*V *× *U*_s_)/[*P*_s_ × (V × U_c_)/P_c_] × 100, where *V* is the urinary flow (mL min^−1^), *U*_s_ is the concentration of solute in urine, *P*_s_ is the concentration of solute in plasma, *U*_c_ is the concentration of creatinine in urine, *P*_c_ is the concentration of creatinine in plasma, and FE_s_ is the fractional excretion of solute (%).

### Renal sampling for RNA and protein extraction

After blood collection, the animals underwent body perfusion with sterile saline (0.9% NaCl) containing heparin (10 U mL^−1^ to avoid clotting) via the left cardiac ventricle. The right femoral vein was sectioned for continuous flow. The right and left kidneys were excised and decapsulated by hand. The renal cortex was dissected using a Sttadie‐Rigg microtome (Thomas Scientific) for total protein and RNA extraction (see below).

### Kidney proximal tubule isolation

The dissected renal cortex from CTRL and DM rats was placed in collagenase B solution (0.125 mg mL^−1^) in Dulbecco's modified Eagle's medium without fetal bovine serum for 30 min at 37°C with agitation. Then, with the aid of a stereoscopic microscope and fine forceps, the proximal tubules were dissected by hand for posterior RNA extraction (Wright et al., 2008).

### RNA extraction

Total RNA from proximal tubules and renal cortex was isolated using a total SV RNA Isolation System (Promega, catalog no. Z3105) following the manufacturer's instructions.

### Real‐time quantitative reverse‐transcription polymerase chain reaction

Total RNA (1 *μ*g) from proximal tubules or renal cortex was reverse transcribed using a RevertAid Premium First Strand cDNA Synthesis Kit (Fermentas, catalog no. K1652) following the manufacturer's instructions.

For qRT‐PCR, the GoTaq 2‐Step RT‐qPCR System (Promega, catalog no. A6010) was used following the manufacturer's instructions. The amplification reactions were performed in 96‐well plates with a final volume of 25 *μ*L using an Eppendorf Mastercycler.

The following parameters were used for all genes studied: 40 cycles at 95°C for 15 sec, 60°C for 1 min. After the cycling program, a dissociation curve (melting curve) was drawn to certify that the amplified product corresponded to the product of interest. Each cDNA was amplified in triplicate. Two negatives controls were used: cDNA minus (PCR reaction without cDNA) and RNA plus (cDNA was replaced by total RNA). The data were analyzed only if both negative controls presented no amplification. mRNA levels were normalized in their respective amplification using 36*β*4 ribosomal gene as the reference gene. The relative mRNA levels were determined by comparing the cycling threshold (CT) PCR among the groups using the following formula: 2^−(ΔΔCt)^, where ΔCt refers to the difference in CT between the mRNA target and the respective reference gene (36B4) CT. The relative change in the levels of target gene mRNA products in the DM group compared with the CTRL group are presented as the mean ± SEM of 2^−(ΔΔCt)^ (Schmittgen and Livak 2008).

The list of primers is given in Table 1.

**Table 1:** Pair of primers used in qRT‐PCR analyses

Sequence ID	Primers	Forward	Reverse	PCR product size (bp)
NM_017106.1	ClC‐5	(701) 5′‐ACGCCTGTGGTTCTGGAATCCCT‐3′ (723)	(819) 5′‐TGACACGGCCAGCACCAAAGT‐3′ (799)	119
NM_030827.1	Megalin	(5036) 5′‐TAGCGATTTGGTTCTCCACC‐3′ (5055)	(5136) 5′‐ACTTGTTGGCCTGCATAACC‐3′ (5117)	101
NM_053332.2	Cubilin	(1030) 5′‐TGCACTCCCGTGGACATCTGTTCG‐3′ (1053)	(1138) 5′‐GAGGACAGGTGCAGACAGGCAAGA‐3′ (1115)	109
NM_031506.1	CFTR	(4226) 5′‐ACTGCTTGATGAGCCTAGTGCC‐3′ (4247)	(4315) 5′‐ACTGTGCAACCAGCGAAGGC‐3′ (4296)	90
NM_022402.2	36B4	(532) 5′‐AATCCTGAGCGATGTGCAG‐3′ (550)	(664) 5′‐GCTGCCATTGTCAAACACC‐3′ (646)	133

### Protein extraction from urine and renal cortex

For urine protein extraction, the urine samples were dialyzed against water using dialysis bags (cutoff 1000 kDa). After dialysis, the urine was lyophilized and resuspended in sample buffer (50 mmol/L Tris‐HCl, 1 mmol/L EDTA, 0.2% SDS, and 1X protease inhibitor cocktail; Roche, 04693116001).

The renal cortex was homogenized in lysis buffer (250 mmol/L sucrose, 20 mmol/L HEPES, 1 mmol/L EDTA, 50 mmol/L NaF, 1 mmol/L phenylmethane sulfonyl fluoride, 1X Roche protease inhibitor cocktail) using a glass Potter homogenizer with a Teflon piston (10 strokes) at ice‐cold temperature. After homogenization, the tissue homogenate was centrifuged at 600*g* for 5 min. The pellet was discarded and the supernatant was transferred to a new tube and centrifuged again at 3000*g* for 10 min.

The protein concentration was determined by the Bradford assay. Total proteins were solubilized in Laemmli sample buffer (Bio‐Rad) with *β*‐mercaptoethanol (1:20) (Souza‐Menezes et al. 2007). After solubilization, 50 *μ*g of protein was subjected to standard SDS‐PAGE using a 4% polyacrylamide stacking gel plus 7.5% polyacrylamide running gel to test lower molecular weight proteins or a 4% polyacrylamide running gel to test higher molecular weight proteins such as megalin and cubilin (>400 kDa).

### Western blot

The SDS‐PAGE running time for the megalin and cubilin proteins was 1 h and 30 min, while the running time for the others proteins (transferrin, ClC5, CFTR, and TNR‐CFTR) was 40 min. After SDS‐PAGE, the proteins were electrophoretically transferred to a polyvinylidene fluoride membrane. The membranes were then blocked with bovine serum albumin 5% in PBS (pH 7.4, block solution). The membranes were then incubated for 12 h at 4°C with commercially available primary antibodies diluted in block solution supplemented with 0.1% Tween‐20 0.1% (PBS‐T): 1:1000 anti‐transferrin (Abcam, AB9538), 1:500 anti‐ClC‐5 (Sigma, C1116), 1:100 anti‐CFTR (R&D Systems, MAB25031), 1:200 anti‐megalin, (Santa Cruz Biotechnology, SC 25470), 1:200 anti‐cubilin (Santa Cruz Biotechnology, SC 20609); and 1:1000 anti‐*β*‐actin (Cell Signaling Technology, 4967). After washing 3 times for 10 min with PBS‐T, the membranes were incubated with their respective secondary antibodies conjugated to peroxidase enzyme for 1 h at room temperature: 1:2000 anti‐goat for transferrin western blot (Santa Cruz Biotechnology, SC2033), 1:5000 anti‐rabbit for ClC‐5 western blot (GE Healthcare, NA934V), 1:1000 anti‐mouse for CFTR western blot (GE Healthcare, NA931V), 1:2000 anti‐rabbit for megalin western blot (GE Healthcare, NA934V), 1:2000 anti‐rabbit for cubilin western blot (GE Healthcare, NA934V), and 1:10,000 anti‐rabbit for *β*‐actin western blot (GE Healthcare, NA934V). Immunodetection of the blots was performed using BM blue POD substrate (Roche, 11484281001). The densitometry of the bands was analyzed using the ImageJ program for Windows. Relative protein expression was determined from the ratio of arbitrary densitometric values for each target to their respective *β*‐actin for renal cortex samples and creatinine mass for urine samples. All control samples were normalized to 1 and the values of the DM samples were related to the controls. It was not observed unspecific bands in CFTR, ClC‐5, megalin, and cubilin as shown in Figure S1.

### Statistical analysis

Data were expressed as means ± SEM. The Shapiro–Wilk normality test was performed to prove the Gaussian distribution of the data. Once the normality condition was satisfied, the data were analyzed by unpaired Student's *t* test using GraphPad Prism 4.0 software (GraphPad, San Diego, CA, USA). Differences were considered significant when *P *<* *0.05.

## Results

### Glycemic and body weight levels

**Figure 2:**
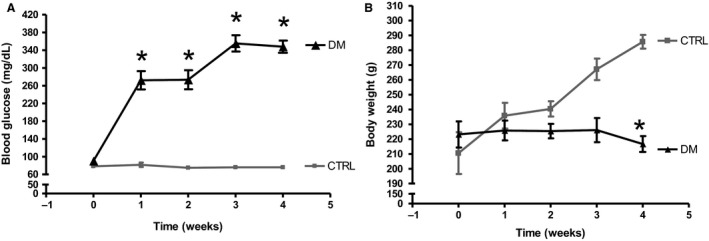
Glycemic levels and body weight of rats in the CTRL and DM groups during the 4 weeks of the experiments. (A) After 5 h fasting, the rats in the DM group showed higher glycemia than those in the CTRL group throughout the experimental timeline. (B) The body weight of DM rats was lower than the CTRL rats (*n *=* *6) in the fourth week of the experiment. All values were compared each week and are presented as means ± SEM. Asterisk (*) indicates a significant difference; CTRL vs. DM (*P *<* *0.05). CTRL,* n *=* *6; DM,* n *=* *8.

Diabetes mellitus is a disorder characterized primarily by high levels of plasma glucose. To verify that the animals used in the experiments had become diabetic, plasma glucose was measured after induction of diabetes and throughout the experimental period. Plasma glucose concentration in the CTRL group remained within the normal range for 1, 2, 3, and 4 weeks after intraperitoneal injection of citrate buffer (81.62 ± 5.0, 74.8 ± 3.3, 75.8 ± 3.6, and 76.0 ±3.1 mg dL^−1^, respectively; *n *=* *6). Rats in the DM group showed increased plasma glucose concentration (mg dL^−1^) for 1, 2, 3, and 4 weeks after induction of type 1 diabetes by intraperitoneal injection of streptozotocin (272.1 ± 20.7, 273.3 ± 21.3, 355.3 ± 18.4, and 348.0 ± 13.7 mg dL^−1^, respectively; *n *=* *8, *P *<* *0.05). These glycemic levels in the DM group are consistent with a type 1 diabetes mellitus scenario without insulin replacement (Fig. 2A).

Because the lack of weight gain is also characteristic of type 1 diabetes, the body weight of the rats was measured over the 4‐week period. Body weight in the DM group remained constant throughout weeks 1–4 (225.8 ± 6.6, 225.4 ± 4.9, 226.1 ± 8.1, and 216.7 ± 5.3 g, respectively; *n *=* *8). Weight gain in the CTRL group became significantly different in the fourth week of the experiment (235.8 ± 8.6, 240.4 ± 5.1, 267.1 ± 7.2, and 285.7 ± 4.6 kg, respectively; *n *=* *6). Thus, the diabetic animals failed to gain weight with age, consistent with the presence of type 1 diabetes mellitus (Fig. 2B).

### Renal function

Renal function was assessed at the end of week 4 using solute clearance and fractional excretion methodologies.

No significant differences in the plasma concentration of sodium, chloride, potassium, creatinine, and total protein were found in DM rats (164.3 ± 9.3 mmol L^−1^, 85.5 ± 1.7 mmol L^−1^, 4.6 ± 0.2 mmol L^−1^, 0.4 ± 0.1 g dL^−1^, and 3.3 ± 0.2 mg dL^−1^, respectively; *n *=* *8, *P *>* *0.05) compared with CTRL rats (175.2 ± 23.7 mmol L^−1^, 91.5 ± 4.6 mmol L^−1^, 4.1 ± 0.3 mmol L^−1^, 0.3 ± 0.1 mg dL^−1^, and 3.3 ± 0.3 g dL^−1^, respectively; *n *=* *6). Plasma glucose concentration was the only solute that was significantly higher in DM rats (368 ± 20.9 mg dL^−1^; *n *=* *8, *P *<* *0.05) compared with CTRL rats (91.2 ± 1.6 mg dL^−1^; *n *=* *6) (Table 2).

**Table 2:** Plasma concentration of sodium, chloride, potassium, creatinine, total protein, glucose, and urea in CTRL and DM groups

Plasma concentration	CTRL	DM
Sodium (mmol/L)	175.2 ± 23.7	164.3 ± 9.3
Chloride (mmol/L)	91.5 ± 4.6	85.5 ± 1.7
Potassium (mmol/L)	4.1 ± 0.3	4.6 ± 0.2
Creatinine (mg/dL)	0.3 ± 0.1	0.4 ± 0.1
Total protein (g/dL)	3.3 ± 0.3	3.3 ± 0.2
Glucose (mg/dL)	91.2 ± 1.6	368 ± 20.9^a^

a Means significant difference from control group.

A significant change in renal function was observed in DM rats compared with CTRL rats. The urinary flow and creatinine clearance were significantly higher in DM rats (0.15 ± 0.0098 mL min^−1^ and 7.1 ± 0.8 mL min^−1^ kg^−1^, respectively; *n *=* *8, *P *<* *0.05) compared with CTRL rats (0.021 ± 0.002 mL min^−1^ and 4.2 ± 0.4 mL min^−1^ kg^−1^, respectively; *n *=* *6). Urinary protein excretion and urinary excretion of albumin were higher in DM rats (18.3 ± 3.1 mg day^−1^ and 10.8 ± 1.4 mg day^−1^, respectively; *n *=* *8, *P *<* *0.05) compared with CTRL rats (9.3 ± 1.1 mg day^−1^ and 1.5 ± 0.2 mg day^−1^, respectively; *n* = 6). The fractional excretion of sodium, chloride, and glucose was higher in DM rats (0.6 ± 0.09%, 1.2 ± 0.2%, and 53.9 ± 5.9%, respectively; *n *=* *8, *P *<* *0.05) compared with CTRL rats (0.3 ± 0.1%, 0.5 ± 0.07%, and 0.2 ± 0.1%, respectively; *n *=* *6). Only the fractional excretion of potassium had no significant difference between CTRL rats (30.4 ± 5.4%; *n *=* *6) and DM rats (39.6 ± 3.8%; *n *=* *8, *P *>* *0.05) (Table 3).

**Table 3:** Renal function parameters in the CTRL and DM groups

Parameters	CTRL	DM
Urinary flow (mL/min/kg)	0.021 ± 0.002	0.15 ± 0.0098^a^
Creatinine clearance (mL/min/kg)	4.2 ± 0.4	7.1 ± 0.8^a^
Urinary protein (mg/day)	9.3 ± 1.1	18.3 ± 3.1^a^
Urinary albumin (mg/day)	1.5 ± 0.2	10.8 ± 1.4^a^
Na^+^ FE (%)	0.3 ± 0.1	0.6 ± 0.09^a^
Cl^−^ FE (%)	0.5 ± 0.07	1.2 ± 0.2^a^
Glucose FE (%)	0.2 ± 0.1	53.9 ± 5.9^a^
K^+^ FE (%)	30.4 ± 5.4	39.6 ± 3.8

a Means significant difference from control group.

### Urinary transferrin analysis

**Figure 3:**
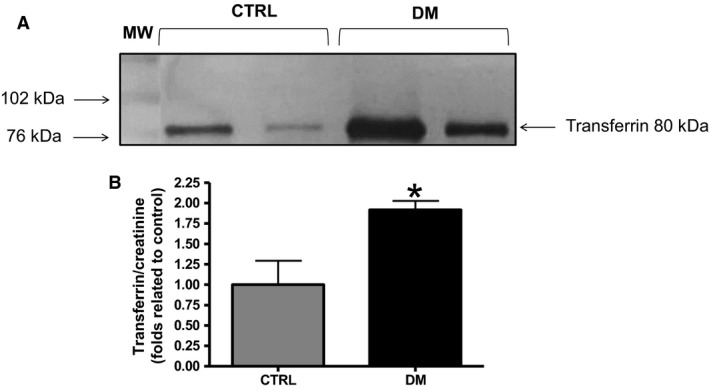
Analyses of transferrin in urine. (A) Western blot analyses of transferrin excretion in the urine of CTRL and DM rats. (B) Densitometric analyses of transferrin excretion in the urine of CTRL and DM rats normalized by their respective creatinine urinary excretion. MW, molecular weight ladder. CTRL,* n *=* *4; DM,* n *=* *4.

Transferrin excretion in urine was analyzed in both groups. DM animals showed a significantly higher excretion of transferrin in urine compared with animals in the CTRL group (Fig. 3A). Densitometric values of western blot bands of transferrin in urine were normalized by their respective creatinine mass on 24‐h excretion. Transferrin excretion in the DM group (1.91 ± 0.1; *n *=* *4, *P *<* *0.05) was found to be about twofold higher that in the CTRL group (1.0 ± 0.29; *n *=* *4) (Fig. 3B). Transferrin is ultrafiltered by the glomerular membrane, but almost completely reabsorbed by receptor‐mediated endocytosis in the proximal tubule, therefore these results suggest a defect in the apical endocytic machinery or changes in the glomerular ultrafiltration membrane.

### Analyses of ClC‐5, CFTR, TNR‐CFTR, megalin, and cubilin protein content in the renal cortex of CTRL and DM rats

**Figure 4:**
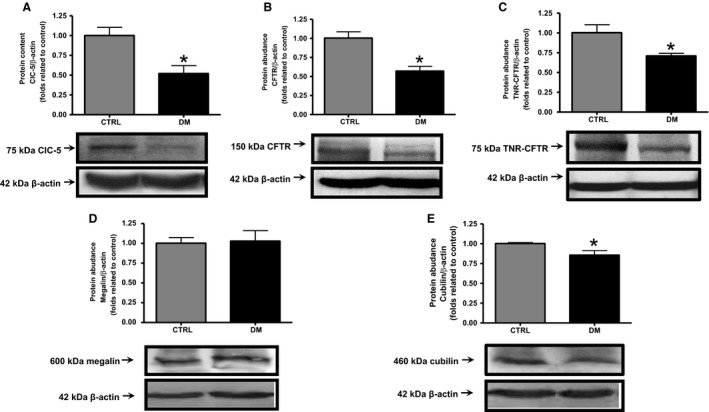
Analyses of ClC‐5, CFTR, TNR‐CFTR, megalin, and cubilin protein content in the renal cortex of CTRL and DM rats. The abundance of key proteins of the endocytic pathway in the renal cortex was measured by western blotting. Graphical representation of the protein content of (A) ClC‐5, (B) CFTR, (C) TNR‐CFTR, (D) megalin, and (E) cubilin. The values are presented as means ± SEM and as fold relative to control. Asterisk (*) indicates a significant difference; CTRL versus DM (*P *<* *0.05). *β*‐Actin expression was used as an internal control. CTRL,* n *=* *6; DM,* n *=* *6.

The protein content (fold related to control) of ClC‐5, CFTR, TNR‐CFTR and cubilin analyzed by western blotting in the renal cortex was significantly lower in DM rats (0.52 ± 0.10, 0.57 ± 0.06, 0.70 ± 0.03, and 0.86 ± 0.06, respectively; *n *=* *6, *P *<* *0.05) compared with CTRL rats (1.00 ± 0.10, 1.00 ± 0.08, 1.00 ± 0.10, and 1.00 ± 0.01, respectively; *n *=* *6) (Fig. 4A, B, C, and E, respectively). No significant change in the content of megalin was observed between DM rats (1.03 ± 0.13; *n *=* *6, *P *>* *0.05) and CTRL rats (1.00 ± 0.07) (Fig. 4D).

### Analyses of ClC‐5, CFTR, megalin, and cubilin mRNA content in the renal cortex of CTRL and DM animals

**Figure 5:**
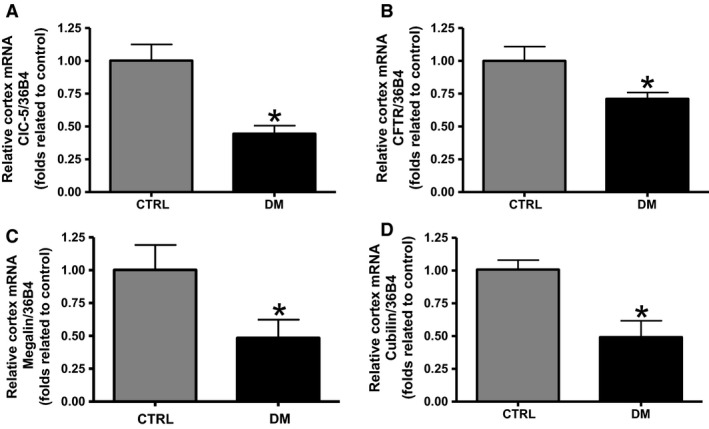
Analyses of ClC‐5, CFTR, megalin, and cubilin mRNA level in the renal cortex of CTRL and DM animals. The mRNA content of key components of the endocytic pathway in the renal cortex was measured by real‐time PCR and 36*β*4 was used as internal control. Graphical representation of the mRNA content of (A) ClC‐5, (B) CFTR, (C) megalin, and (D) cubilin in the renal cortex of CTRL and DM rats. The values are presented as means ± SEM and as fold relative to control. Asterisk (*) indicates a significant difference; CTRL vs. DM (*P *<* *0.05). CTRL,* n *=* *4; DM,* n *=* *7.

The relative mRNA content of ClC‐5, CFTR, megalin, and cubilin, analyzed by semiquantitative real‐time PCR in the renal cortex, was significantly lower in DM rats (0.45 ± 0.06, 0.71 ± 0.05, 0.48 ± 0.14, and 0.49 ± 0.13, *n *=* *7, respectively; *P *<* *0.05) compared with CTRL rats (1.00 ± 0.12, 1.00 ± 0.11, 1.00 ± 0.19, and 1.00 ± 0.07, respectively; *n *=* *4) (Fig. 5A–D).

### Analyses of ClC‐5, CFTR, megalin, and cubilin mRNA level in dissected proximal tubule of CTRL and DM animals

**Figure 6:**
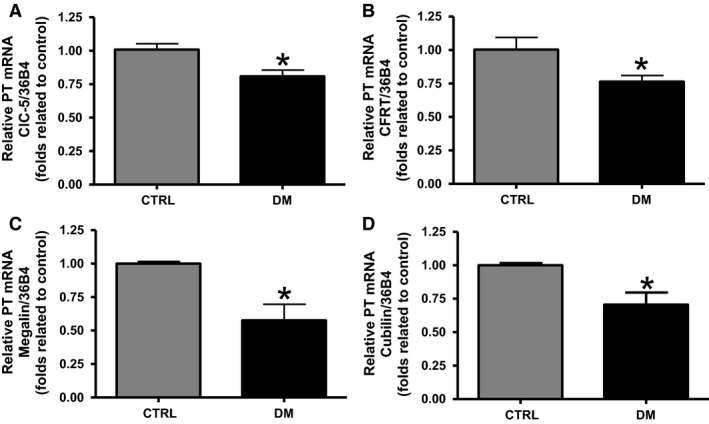
Analyses of ClC‐5, CFTR, megalin, and cubilin mRNA level in dissected proximal tubule of CTRL and DM rats. The mRNA content of key components of the endocytic pathway in the proximal tubule was measured by real‐time PCR and 36B4 was used as internal control. Graphical representation of the mRNA content of (A) ClC‐5, (B) CFTR, (C) megalin, and (D) cubilin in the proximal tubule of CTRL and DM rats. The values are presented as means ± SEM and as fold relative to control. Asterisk (*) indicates a significant difference; CTRL versus DM (*P *<* *0.05). CTRL,* n *=* *4; DM,* n *=* *4.

In isolated proximal tubule of DM rats, lower relative mRNA content of ClC‐5, CFTR, megalin, and cubilin (0.81 ± 0.05, 0.76 ± 0.05, 0.58 ± 0.12, and 0.71 ± 0.09, respectively; *n *=* *4, *P *<* *0.05) was observed compared with CTRL rats (1.00 ± 0.04, 1.00 ± 0.09, 1.00 ± 0.02, and 1.00 ± 0.02, respectively; *n *=* *4) (Fig. 6).

## Discussion

The present work shows that diabetes induced changes in proximal tubule that may be an important player in the development of microalbuminuria and proteinuria in diabetic kidney disease.

The main pathophysiological characteristic of diabetes mellitus is the presence of a high concentration of plasma glucose. In type 1 diabetes, this is a consequence of destruction of pancreatic *β* cells, decreasing the production of insulin (Voltarelli et al. 2007).

In animal models, the injection of STZ (45 mg kg^−1^) to induce type 1 DM is a well‐established model known to reduce the production of insulin without the need for exogenous administration of this hormone for animal survival (Castiglione et al. 2013; Betz and Conway 2014). A single dose of STZ is not toxic to the kidney or to the proximal tubule, as shown previously by Palm et al. (2004). The authors transplanted *β* cells to eliminate diabetes in the STZ‐treated animals and found that albumin excretion in urine of this group went back to normal ranges after the transplantation compared with STZ‐treated animals without *β*‐cell transplant. This indicates that albuminuria is a consequence of diabetes and not an effect of STZ per se (Palm et al. 2004). Therefore, the single dose of STZ mentioned above was used in the present work and, after administration, a hyperglycemic state above 250 mg dL^−1^ confirmed that diabetes was successfully induced. Moreover, STZ‐induced diabetic animals showed increased fractional excretion of glucose compared with animals in the control group, suggesting saturation of the mechanisms of glucose transport in renal tubules. Maintenance of a high glucose load along the whole length of the renal tubules stimulates osmotic diuresis, with increased urinary flow (Bardoux et al. 2001; Katsuno et al. 2007). There was an increase in urinary flow in the diabetic animals in this study compared with the control animals, in agreement with other studies; this is probably induced by an osmotic effect of the high glucose concentration in tubular lumen and increased glomerular filtration rate (GFR) observed in the DM group (DiPetrillo et al. 2003; Rebsomen et al. 2006; Dronavalli et al. 2008; Castiglione et al. 2013).

GFR is commonly estimated by measuring creatinine clearance (Moresco et al. 2013). The increased creatinine clearance observed in this study is in agreement with several other studies that correlated with DN (Hiragushi et al. 2004; Samnegård et al. 2005; Ichinose et al. 2007; Dronavalli et al. 2008).

As mentioned previously, DM animals were found to have a higher fractional excretion of glucose; in addition, increased fractional excretion of sodium and chloride was observed compared with controls with no changes in the plasma concentration of these solutes. Higher fractional excretion of sodium and chloride was described previously in patients and in experimental animals with diabetes mellitus and it was suggested to be related to a tubular disability in handling these solutes. Furthermore, it positively correlated with an increased rate of urine flow, which was observed in the DM animals in this study (Fiorina et al. 2003; Song et al. 2003; Castiglione et al. 2013).

Several others important characteristics are present in DN. Microalbuminuria and proteinuria are classic and important biomarkers of this disease (Ichinose et al. 2007; Dronavalli et al. 2008). In the present study, the DM rats showed higher daily excretion of albumin and total protein compared with control rats.

Loss of protein in urine is known to occur due to increased permeability of the glomerulus capillary wall and/or decreased proximal tubule endocytosis. Participation of the proximal tubule in diabetes proteinuria has become a focus of interest in recent years and has been shown to be more than a simple downstream factor induced by glomerular injury (Gilbert and Cooper 1999; Ichinose et al. 2007; Dronavalli et al. 2008; Tang et al. 2011). To provide insight into the tubular mechanisms underlying DN, transferrin excretion in urine was analyzed in DM and CTRL animals. Transferrin is a 75‐kDa protein that is ultrafiltered by the glomerulus and largely reabsorbed by proximal tubule via receptor‐mediated endocytosis. Transferrin binds to its receptor cubilin to be endocytosed (Kozyraki et al. 2001; Jeong et al. 2014). In the present work, DM animals excreted about twofold more transferrin in urine compared with CTRL animals. This result suggests proximal tubule dysfunction, more specifically a defect in the endocytic machinery, but only based on the data, we cannot discount hyperfiltration of transferrin.

To better understand diabetic proximal tubule endocytosis dysfunction, the protein and mRNA content of tubular endocytic components (such as ClC‐5, CFTR, TNR‐CFTR, megalin, and cubilin) of diabetic animals were analyzed.

The protein content of ClC‐5, CFTR, TNR‐CFTR, and cubilin were found to be lower in the renal cortex of DM animals compared with CTRL animals. No changes were detected between both groups in the protein content of megalin. The mRNA content of ClC‐5, CFTR, megalin, and cubilin was also evaluated and was observed to be lower in the renal cortex of DM animals compared with control animals.

The pattern of protein content of megalin in renal cortex analyzed in this work is different from that observed for its mRNA content. This result suggests that megalin mRNA and protein content in the renal cortex of diabetic rats may be regulated differently. This differential regulation of megalin mRNA and protein has already been reported in ClC‐5 KO mice, but in the opposite way. This pattern of independent protein regulation of megalin and cubilin has been demonstrated in the same animal model (Christensen et al. 2003). Additional experiments are necessary to understand the differential regulation of megalin and cubilin protein and mRNA expression. Furthermore, a lack of validation of the anti‐megalin antibody is a limitation of this study; however, megalin single band demarcations were localized in the expected molecular weight size (Figure S1) plus other studies had successfully used the same antibody previously (Girardi et al. 2008; Gressner et al. 2008; Tsaroucha et al. 2008; Prutskova and Seliverstova 2013).

Several previous works have shown that ClC‐5 and CFTR are important components in the proximal tubule endocytic apparatus and revealed their association with other important players, such as megalin and cubilin receptors (Kozyraki et al. 2001; Jentsch 2005; Jouret et al. 2007; Souza‐Menezes et al. 2007; Jouret and Devuyst 2009; Carraro‐Lacroix et al. 2010). TNR‐CFTR is a splice variant form of CFTR that is far more prevalent in the kidney than the usual form. It has been suggested that TNR‐CFTR function in the kidney, despite being poorly understood, is involved in proximal tubule receptor‐mediated endocytosis (Tojo et al. 2001, 2003; Souza‐Menezes et al. 2014). Our findings are in agreement with these studies, however, this is the first time that endocytic proteins have been analyzed in the context of diabetes.

In addition, as the reabsorption of LMW proteins and albumin is known to occur mainly in the proximal tubule, a fine technique was used to dissect this part of the nephron from the renal cortex. The mRNA content of all endocytic components, ClC‐5, CFTR, megalin, and cubilin was found to be lower in dissected proximal tubule of DM animals compared with control animals. These data are in agreement with that observed for mRNA content of ClC‐5, CFTR, megalin, and cubilin in renal cortex.

Megalin and cubulin are also expressed in glomerular cells (Prabakaran et al. 2011, 2012; Ceol et al. 2012). Because the mRNA content of these receptors follows the same pattern in renal cortex and isolated proximal tubule, we can suggest that the mRNA content of megalin and cubilin from glomerular cells does not contribute in a significant way to the downregulation in mRNA content observed in renal cortex of DM rats.

To our knowledge, this is the first time that the protein content of ClC‐5, CFTR, cubilin, and TNR‐CFTR has been shown to have lower expression in the kidney of diabetic rats after 4 weeks. For the first time, the mRNA content of these components (ClC‐5, CFTR, cubilin, and megalin) was also found to be downregulated in the renal cortex and specifically in the proximal tubule of DM animals. Thus, these data provide an important new insight into the pathophysiology of DN.

Several explanations may account for the downregulation of these proteins in the diabetic kidney. A recent work from Peruchetti et al. (2014) showed that overload of albumin in the proximal tubule may be one of the reasons. The authors showed that megalin knockdown and overload of albumin in proximal tubule cells have very similar effects. In both situations, there is an inhibition of PKB phosphorylation and activation of the PI3K/mTORC2/PKB/mTORC1/S6 kinase (S6K) pathway (Peruchetti et al. 2014). The pathophysiological role of albumin overload was also shown in different experimental models (Hryciw et al. 2004; Caruso‐Neves et al. 2006). Thus, the higher excretion of albumin that we found in DM animals might be one of the factors involved in the downregulation of endocytic machinery components encountered in the present work.

Furthermore, PI3K, a common intracellular downstream effector of glucose and insulin, was shown to be associated with CFTR activation (Blachly and Baiocchi 2014). Blachly and Baiocchi (2014) showed that PI3K is involved in the modulation of CFTR by insulin in kidney cells, highlighting a possible association between the lack of insulin in the STZ‐induced diabetic animal model and CFTR dysfunction. Nevertheless, more studies on the complex relationship between the endocytic machinery components and diabetes features, such as hyperglycemia, lack of insulin, and albumin overload, are indeed necessary (Blachly and Baiocchi 2014).

Regarding the excretion of transferrin, the higher amount encountered in the urine of DM animals may be associated with the downregulation of the endocytic components mentioned earlier. A previous work from Jouret et al. (2007) also highlighted the association of higher urinary transferrin excretion with CFTR downregulation. These authors found a greater amount of transferrin in the urine of patients with cystic fibrosis, a genetic disease that is characterized by CFTR dysfunction (Jouret et al. 2007). Also, this increase in urinary excretion of transferrin was observed in ClC‐5 KO mice (Christensen et al. 2003).

In conclusion, for the first time, the present study shows lower expression of different components of the endocytic apparatus (such as ClC‐5, CFTR, TNR‐CFTR, and cubilin) in the renal cortex and in the proximal tubule of early‐stage type 1 diabetic rats. The present data suggest a correlation between proximal tubular endocytosis dysfunction and the higher urinary excretion of total protein, albumin, and transferrin observed in the DM animals. Moreover, these results provide insights into the critical role of the proximal tubule in DN pathophysiology, thus highlighting an important pathway for the future development of tools for the diagnosis and intervention in the course of diabetic kidney disease.

## Conflict of Interest

The authors declare no competing financial interests.

## Data Accessibility

## Supporting information




**Figure S**1**.** Full blot of ClC‐5, CFTR, TNR‐CFTR, megalin, and cubilin. Representative images of ClC‐5 (A), CFTR and TNR‐CFTR (B), megalin (C), and cubilin (D) full blots. CTRL = control rats. DM = diabetic rats. MW = molecular weight marker (ECL^™^ Rainbow^™^ Marker ‐ Full Range, Amersham^™^, Code: RPN800E). The SDS‐PAGE running of Megalin and Cubilin proteins was for 1 h and 30 min, after this time, the 225 kDa MW band was just out of the membrane and for this reason the MW bands can not been seen in C and D.
