# Supplementary material for: Diabetic rats present higher urinary loss of proteins and lower renal expression of megalin, cubilin, ClC‐5, and CFTR
Source: Physiol Rep. 2017 Jul 4;5(13):e13335. doi: 10.14814/phy2.13335 (PMC5506523; doi:10.14814/phy2.13335)
Supplement: Supplementary file 1 — Figure S1. Full blot of ClC‐5, CFTR, TNR‐CFTR, megalin, and cubilin. Representative images of ClC‐5 (A), CFTR and TNR‐CFTR (B), megalin (C), and cubilin (D) full blots. CTRL = control rats. DM = diabetic rats. MW = molecular weight marker (ECL™ Rainbow™ Marker ‐ Full Range, Amersham™, Code: RPN800E). The SDS‐PAGE running of Megalin and Cubilin proteins was for 1 h and 30 min, after this time, the 225 kDa MW band was just out of the membrane and for this reason the MW bands can not been seen in C and D. [file PHY2-5-e13335-s001.pptx]

## Slide 1
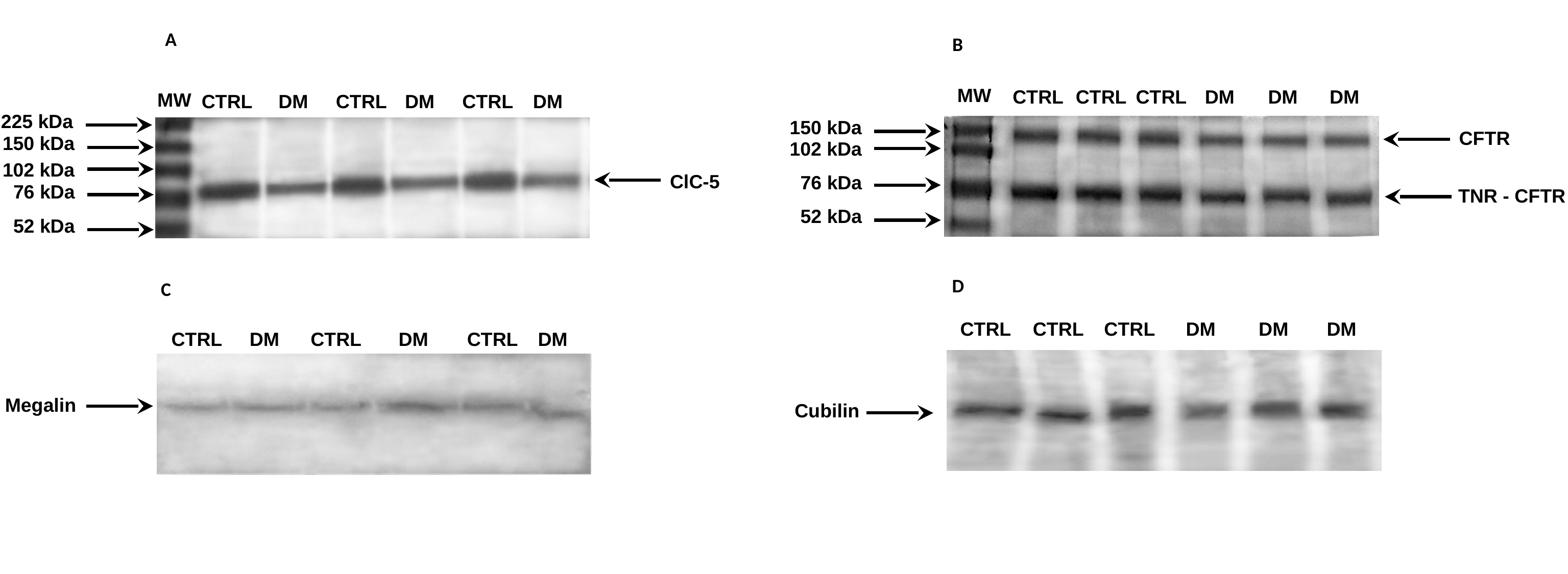

A
B
MW
CTRL
CTRL
CTRL
DM
DM
DM
MW
CTRL
DM
CTRL
DM
CTRL
DM
225 kDa
150 kDa
CFTR
150 kDa
102 kDa
102 kDa
ClC-5
76 kDa
76 kDa
TNR - CFTR
52 kDa
52 kDa
D
C
CTRL
CTRL
CTRL
DM
DM
DM
CTRL
DM
CTRL
DM
CTRL
DM
Megalin
Cubilin
